# The Pathogenic Bacteria of Deep Neck Infection in Patients with Type 1 Diabetes, Type 2 Diabetes, and Without Diabetes from Chang Gung Research Database

**DOI:** 10.3390/microorganisms9102059

**Published:** 2021-09-29

**Authors:** Chih-Wei Luan, Chia-Yen Liu, Yao-Hsu Yang, Ming-Shao Tsai, Yao-Te Tsai, Cheng-Ming Hsu, Ching-Yuan Wu, Pey-Jium Chang, Geng-He Chang

**Affiliations:** 1 Department of Otorhinolaryngology-Head and Neck Surgery, Lo Sheng Sanatorium and Hospital Ministry of Health and Welfare, New Taipei City 24257, Taiwan; jackluan2010@gmail.com; 2 Department of Otolaryngology, Chang Gung Memorial Hospital, Chiayi 61363, Taiwan; b87401061@cgmh.org.tw (M.-S.T.); yaote1215@gmail.com (Y.-T.T.); scm00031@gmail.com (C.-M.H.); 3 Graduate Institute of Clinical Medical Sciences, College of Medicine, Chang Gung University, Taoyuan 33302, Taiwan; smbepigwu77@gmail.com (C.-Y.W.); peyjiumc@mail.cgu.edu.tw (P.-J.C.); 4 Health Information and Epidemiology Laboratory, Chang Gung Memorial Hospital, Chiayi 61363, Taiwan; qchiayen@gmail.com (C.-Y.L.); r95841012@ntu.edu.tw (Y.-H.Y.); 5 Department of Traditional Chinese Medicine, Chang Gung Memorial Hospital, Chiayi 61363, Taiwan; 6 School of Traditional Chinese Medicine, College of Medicine, Chang Gung University, Taoyuan 33302, Taiwan; 7 Faculty of Medicine, College of Medicine, Chang Gung University, Taoyuan 33302, Taiwan

**Keywords:** cervical abscess, cervical cellulitis, database, hyperglycemia, Diabetes, Klebsiella pneumoniae

## Abstract

Deep neck infection (DNI) is a lethal emergent condition. Patients with types 1 and 2 diabetes mellitus (T1DM and T2DM, respectively) are predisposed to DNI and have poorer prognoses. The mainstay of the treatment is surgical drainage and antibiotics; however, the pathogenic bacteria of T1DM-DNI have not been studied before. We obtained the data of 8237 patients with DNI who were hospitalized from 2004 to 2015 from the Chang Gung Research Database, which contains multi-institutional medical records in Taiwan. Using diagnostic codes, we classified them into T1DM-DNI, T2DM-DNI, and non-DM-DNI and analyzed their pathogenic bacteria, disease severity, treatment, and prognosis. The top three facultative anaerobic or aerobic bacteria of T1DM-DNI were *Klebsiella pneumoniae* (KP, 40.0%), *Viridans Streptococci* (VS, 22.2%), and methicillin-sensitive *Staphylococcus aureus* (MSSA, 8.9%), similar for T2DM (KP, 32.2%; VS, 23.3%; MSSA, 9.5%). For non-DM-DNI, it was VS (34.6%), KP (9.8%), and coagulase-negative *Staphylococci* (8.7%). The order of anaerobes for the three groups was *Peptostreptococcus micros*, *Prevotella intermedia*, and *Peptostreptococcus anaerobius*. Patients with T1DM-DNI and T2DM-DNI had higher white blood cell (WBC) counts and C-reactive protein (CRP) levels, more cases of surgery, more cases of tracheostomy, longer hospital stays, more mediastinal complications, and higher mortality rates than those without DM-DNI. Patients in the death subgroup in T1DM-DNI had higher WBC counts, band forms, and CRP levels than those in the survival subgroup. Patients with DM-DNI had more severe disease and higher mortality rate than those without DM-DNI. KP and *Peptostreptococcus micros* are the leading pathogens for both patients with T1DM-DNI and those with T2DM-DNI. Clinicians should beware of high serum levels of infection markers, which indicate potential mortality.

## 1. Introduction

Deep neck infection (DNI) is a common, life-threatening infectious disease that is usually encountered at the emergency department and requires aggressive treatment. Past studies have reported that type 2 diabetes mellitus (T2DM) is a risk factor for DNI and could lead to higher mortality, longer hospital stays, and many complications compared with non-DM patients [1,2]. Our research also proved that type 1 diabetes mellitus (T1DM) is one of the risk factors for DNI (adjusted hazard ratio: 10.7, *p* < 0.001) and is associated with a significantly longer hospital stay than non-DM DNI [3]. Diabetes-related immunosuppression is considered a possible cause [4].

Because DNIs are infectious emergencies, the diseases sometimes progress rapidly and even cause complications such as mediastinal abscess, which will greatly increase the mortality rate [5]. Therefore, in addition to timely incision and debridement, the adequate choice of empirical antibiotic is important before obtaining the results of bacterial cultures [6]. Past studies have found the most common pathogen of the DNI is *Viridans streptococci* (VS) in general, but for patients with T2DM, *Klebsiella* *pneumoniae* (KP) is the leading strain [7,8]. The discovery provided clinicians with an important reference when choosing empirical antibiotics for DNI treatment. However, in the past, there has been no relevant research on the pathogenic strain of DNI in patients with T1DM. Because of the lack of laboratory data on bacterial cultures in the national database we used before to confirm T1DM is a risk for DNI, it is impossible to further analyze the pathogenic bacteria of T1DM-DNI as a reference for clinical antibiotic selection. In addition, it is remarkably difficult to collect enough T1DM-DNI patients in a single medical institution for an analysis of the pathogenic bacteria of T1DM-DNI. Herein, the use of a multi-institutional database is an important way to make the research possible. We used a multi-medical institutional database in Taiwan, the Chang-Gung Research Database (CGRD), which contains a large number of original medical records, to investigate significant information on DNI in patients with T1DM, T2DM, and non-DM, including the pathogenic bacterial speculum, disease manifestation, and prognosis.

## 2. Materials and Methods

### 2.1. Data Source—The CGRD

The CGRD is a de-identified database derived from the medical records of the Chang Gung Memorial Hospital (CGMH), and it is systematically updated annually to include new data generated in the CGMH. The CGMH, which was founded in 1976, is currently the biggest hospital system in Taiwan, and it comprises seven medical institutes, which are located from the northeast to southern regions of Taiwan: these include Keelung CGMH, Taipei CGMH, Linkou CGMH, Taoyuan CGMH, Yunlin CGMH, Chiayi CGMH, and Kaohsiung CGMH. The CGMH has a total of 10,070 beds and admits more than 280,000 patients each year [9]. In these decades, the CGMH supported and promoted clinical and scientific studies, and, by the year 2019, more than 2900 articles had been published in a diverse range of reputed journals by the CGMH staff. Some of these studies were multicenter studies carried out in the different centers of the CGMH with relatively large sample sizes.

We conducted the study in accordance with the guidelines of the Declaration of Helsinki. The requirement for participants’ informed consent was waived because all the data collected in this study were de-identified and the study neither violated its participants’ rights nor adversely affected their welfare. The study was approved by the Institutional Review Board (IRB) of the CGMH (IRB number: 201900476B0C501) in 8 August 2019.

### 2.2. Study Groups—DNI in Patients with DM

**Figure 1:**
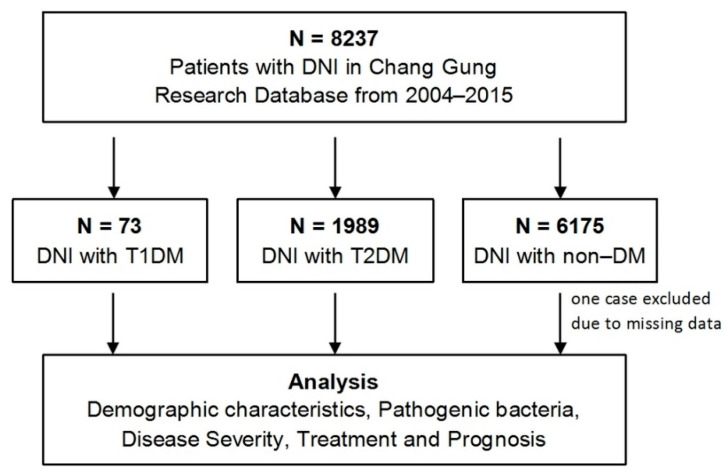
Enrolment and classification schema of DNI cases. Abbreviations: DNI, deep neck infection; T1DM, type 1 diabetes mellitus; T2DM, type 2 diabetes mellitus.

We extracted data on inpatient DNI from the database of the CGRD from 1 January 2004, to 31 December 2015, by using DNI-related diagnostic Classification of Diseases 9 codes (ICD9): 528.3 (cellulitis and abscess of oral soft tissues; Ludwig angina), 478.22 (parapharyngeal abscess), 478.24 (retropharyngeal abscess), and 682.11 (cellulitis and abscess of the neck). There are five inpatient diagnoses in the system, and we only filtered the cases whose main diagnosis was DNI. Further, we used the ICD-9 codes of T1DM (250.01, 250.03, 250.11, 250.13, 250.21, 250.23, 250.31, 250.33, 250.41, 250.43, 250.51, 250.53, 250.61, 250.63, 250.71, 250.73, 250.81, 250.83, 250.91, and 250.93) and T2DM (250.0, 250.00, 250.02, 250.1, 250.10, 250.12, 250.4, 250.40, 250.42, 250.5, 250.50, 250.52, 250.6, 250.60, 250.62, 250.7, 250.70, 250.72, 250.9, 250.90, and 250.92) to separate those DNI cases into three groups (T1DM-DNI, T2DM-DNI, and non-DM-DNI) and analyzed their bacterial strains, disease manifestations, methods of therapy, and prognosis (Figure 1).

### 2.3. Comorbidities

Comorbidities include the risk factors for DNI identified in previous studies [3,5,10,11,12] and basic medical comorbidities. The following comorbidities were defined using ICD-9-CM codes recorded in the claims data: end-stage renal disease, ESRD (ICD-9-CM code: 585, 586, 403.01, 403.11, 403.91, 404.02, 404.03, 404.12, 404.13, 404.92, and 404.93); liver cirrhosis, LC (ICD-9-CM codes: 571.2 and 571.5–571.6); systemic autoimmune disease (ICD-9-CM codes: 443.1, 446.0, 446.2, 446.4–446.5, 446.7, 696.0–696.1, 710.0–710.4, and 714.0–714.4); hypertension, HTN (ICD-9-CM codes: 401–405); cerebrovascular accident, CVA (ICD-9-CM codes: 430–438); coronary artery disease, CAD (ICD-9-CM codes: 410–414); and chronic obstructive pulmonary disease, COPD (ICD-9-CM codes: 491, 492, and 496) [3,5,10,11,12]. Medical comorbidities were included if they appeared at least once in the diagnoses of inpatients or at least thrice in the diagnoses of outpatients. Comorbidities were included if they occurred within 12 months before the DNIs.

### 2.4. Bacterial Spectrum

We identified and analyzed the results of “pus” and “blood” cultures in those DNI patients and determined whether there was complicated bacteremia based on the results of blood cultures. In general, facultative anaerobes, such as *Streptococcus* species, *Staphylococcus* species, and *Klebsiella* species, and aerobes, such as *Pseudomonas* species, are considered major pathogenic bacteria for DNI. Clinically, empirical antibiotics usually aim to cover the most common pathogens of facultative anaerobes or aerobes. Herein, the types of pathogenic bacteria were classified into three groups, namely (1) facultative anaerobes, such as *Staphylococcus* species and aerobic bacteria, such as *Pseudomonas* species, (2) anaerobic bacteria, such as *Peptostreptococcus* species, and (3) fungi. Each species composed of a variety of bacteria, for example, *Staphylococcus* species containing *Staphylococcus aureus*, *Staphylococcus epidermidis*, *Coagulase negative staphylococcus*, etc. We ranked the top three “species” and “single bacteria” of facultative anaerobes or aerobes, anaerobes and fungi in T1DM-DNI, T2DM-DNI and non-DM-DNI.

### 2.5. Therapeutic Classification

We assessed the therapeutic methods used for treating patients with DNI. The treatment methods were divided into two subgroups: antibiotics with or without incision and drainage. These interventions, including abscess aspiration and surgical debridement, were identified using the claims records during hospitalization for DNI treatment.

### 2.6. Disease Severity and Prognosis Evaluation

To evaluate clinical manifestations and disease prognosis, we analyzed the laboratory data, performance of tracheostomy, duration of hospital stays, admission in intensive care units (ICUs), occurrence of mediastinitis, and mortality rate in those DNIs. Mortality was defined as death occurring during DNI treatment. Regarding the relationship between the overall survival of all DNI patients and the severity of disease, we applied a case-control analysis to investigate the odd ratios of various variables to death. In addition, we further analyzed the mortality and survival cases of T1DM and T2DM to evaluate the difference in DNI performance between the two groups.

### 2.7. Statistical Analysis

The sociodemographic data and comorbidities of patients with DNI were compared between the T1DM, T2DM, and non-DM groups using Pearson’s chi-squared test and Fisher’s exact test. The therapeutic methods and percentage of ICU admission in those three groups were compared using the Fisher exact test, which was also used to compare the incidence of tracheostomy, surgery, bacteremia, mediastinitis, and mortality. The duration of hospital stays and laboratory results were compared using the Kruskal–Wallis test. All the analyses were performed using SAS software version 9.4 (SAS Institute, Cary, NC, USA), and the threshold for statistical significance was set at *p* < 0.05.

## 3. Results

### 3.1. DNI Population

The data collection procedure in this study is presented in Figure 1. According to the ICD diagnostic codes, from 1 January 2004, to 31 December 2015, a total of 8237 patients who were hospitalized for DNI were identified from the CGRD. Then, according to the diagnosis code of DM, those patients with DNI were classified into groups of 73 patients with T1DM-DNI, 1989 patients with T2DM-DNI, and 6175 patients with non-DM-DNI (1 patient was excluded because of missing data).

### 3.2. Sociodemographic Data and Comorbidities

Table 1 illustrates the distribution of sociodemographic characteristics and medical comorbidities identified in the T1DM, T2DM, and non-DM groups. In all the three groups, there was significantly more men than women (T1DM vs. T2DM vs. non-DM: 53.4% vs. 64.9% vs. 66.8%, *p* = 0.02) and a significantly high proportion of people aged <65 years (<65: 69.9% vs. 63.2% vs. 84.8%, *p* < 0.001). Patients with DM-DNI had significantly more medical comorbidities of ESRD (11.0% vs. 10.0% vs. 2.2%, *p* < 0.001), LC (9.6% vs. 6.7% vs. 2.5%, *p* < 0.001), systemic autoimmune diseases (1.4% vs. 3.2% vs. 2.0%, *p* < 0.001), HTN (45.2% vs. 54.2% vs. 16.2%, *p* = 0.01), CVA (16.4% vs. 16.2% vs. 4.8%, *p* < 0.001), CAD (11.0% vs. 12.7% vs. 3.2%, *p* < 0.001), and COPD (9.6% vs. 8.0% vs. 4.2%, *p* < 0.001) than patients with non-DM-DNI.

### 3.3. Bacterialculture Spectrum

We analyzed the bacterial culture records of these patients with DNI. The bacterial cultures were performed in 90.4% of patients with T1DM-DNI, 79.8% of patients with T2DM-DNI, and 76.2% of patients with non-DM-DNI, and the positive culture rates of patients with T1DM-DNI, T2DM-DNI, and non-DM-DNI were 68.2%, 57.1%, and 42.6%, respectively. According to culture results, the bacterial spectra in the three groups were classified into (1) facultative anaerobes or aerobes, (2) anaerobes, and (3) fungi, and detailed information is provided in the Supplementary Data (Supplementary Table).

#### 3.3.1. Top Three Species of Facultative Anaerobes and Aerobes

**Figure 2:**
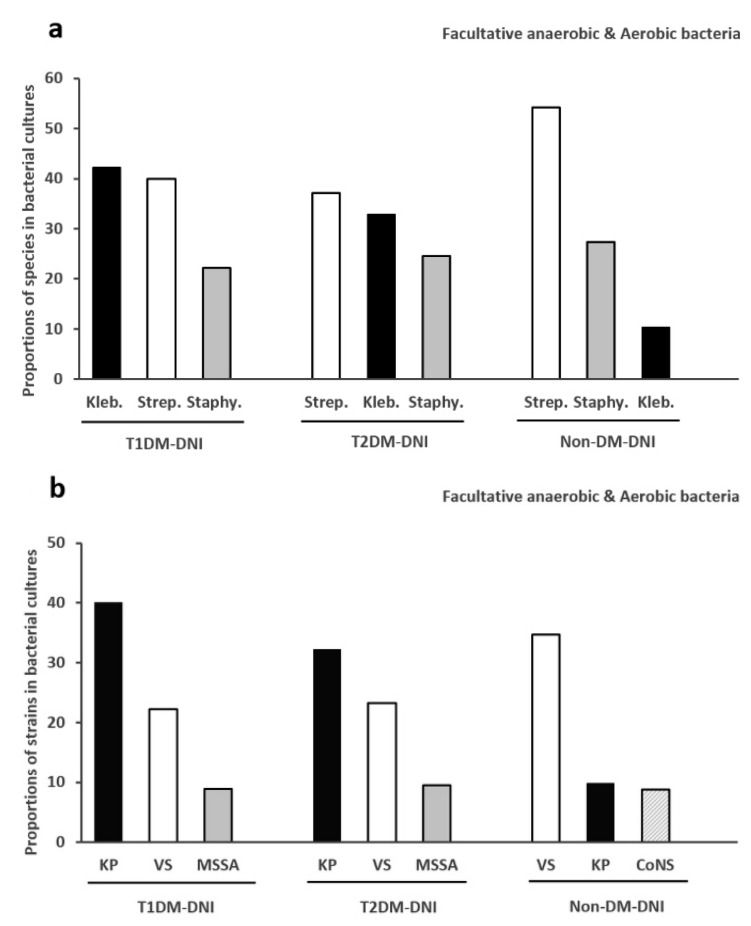
(**a**): Top three species of facultative anaerobes and aerobes in T1DM-DNI, T2DM-DNI, and non-DM-DNI. (**b**): Top three bacteria of facultative anaerobes and aerobes in T1DM-DNI, T2DM-DNI, and non-DM-DNI. Abbreviations: Kleb., Klebsiella species; Strep., Streptococcus species; Staphy., Staphylococcus species. KP, Klebsiella pneumoniae; VS, Viridians Streptococci; MSSA, methicillin-sensitive Staphylococcus aureus; CoNS, coagulase-negative Staphylococcus. DNI, deep neck infection; T1DM, type 1 diabetes mellitus; T2DM, type 2 diabetes mellitus.

Figure 2a shows the top three species (sp.) of facultative anaerobic or aerobic bacteria in the three groups. The top three species of T1DM-DNI are *Klebsiella* sp. (42.2%), *Streptococcus* sp. (40.0%), and *Staphylococcus* sp. (22.2%). The top three species of T2DM-DNI are *Streptococcus* sp. (37.2%), *Klebsiella* sp. (32.9%), and *Staphylococcus* sp. (24.6%). Among non-DM-DNI, the top three species are *Streptococcus* sp. (54.3 %), *Staphylococcus* sp. (27.3%), and *Klebsiella* sp. (10.4%).

#### 3.3.2. Top Three Bacteria of Facultative Anaerobes and Aerobes

Figure 2b shows the top three bacteria of facultative anaerobes or aerobes in each group. The leading pathogens are KP (40.0%), VS (22.2%), and methicillin-sensitive *Staphylococcus aureus* (MSSA; 8.9%) in T1DM-DNI. The top three pathogens in T2DM-DNI are KP (32.2%), VS (23.3%), and MSSA (9.5%). The top three pathogens in non-DM-DNI are VS (34.6%), KP (9.8%), and coagulase-negative *Staphylococci* (CoNS; 8.7%).

#### 3.3.3. Top Three Species of Anaerobes

**Figure 3:**
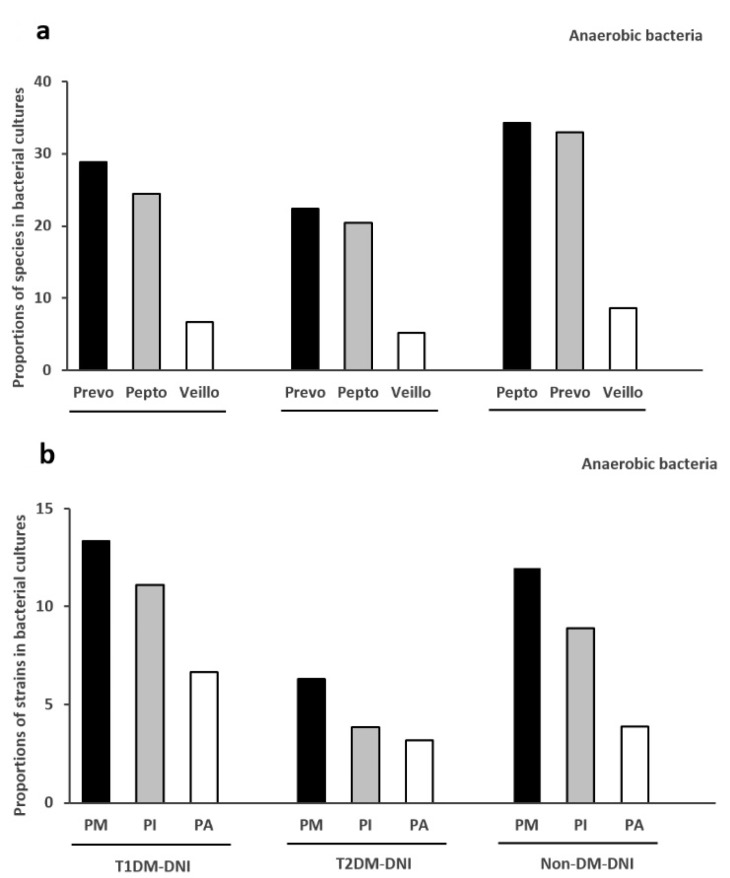
(**a**): Top three species of anaerobes in T1DM-DNI, T2DM-DNI, and non-DM-DNI, (**b**): Top three anaerobic bacteria in T1DM-DNI, T2DM-DNI, and non-DM-DNI. Abbreviations: Prevo, *Prevotella* species; Pepto, *Peptostreptococcus* species; Veillo, *Veillonella* species; PM, *Peptostreptococcus micros*; PI, *Prevotella intermedia*; PA, *Peptostreptococcus anaerobius*. DNI, deep neck infection; T1DM, type 1 diabetes mellitus; T2DM, type 2 diabetes mellitus.

Figure 3a shows the top three species of anaerobes in the three groups. The top three species of T1DM-DNI are *Prevotella* sp. (28.9%), *Peptostreptococcus* sp. (24.4%), and *Veillonella* sp. (6.7%). The top three anaerobic species of T2DM-DNI are *Prevotella* sp. (22.4%), *Peptostreptococcus* sp. (20.4%), and *Veillonella* sp. (5.2%). The three leading anaerobes in non-DM-DNI are *Peptostreptococcus* sp. (34.2%), *Prevotella* sp. (32.9%), and *Veillonella* sp. (8.7%).

#### 3.3.4. Top Three Anaerobic Bacteria

Figure 3b shows the top three anaerobic bacteria in the three groups. The top three anaerobes in T1DM-DNI are *Peptostreptococcus micros* (PM; 13.3%), *Prevotella intermedia* (PI; 11.1%), and *Peptostreptococcus anaerobius* (PA; 6.7%). In T2DM-DNI, the top three anaerobes are PM (6.3%), PI (3.9%), and PA (3.2%); in non-DM-DNI, the top three anaerobes are PM (12.0%), PI (8.9%), and PA (3.9%).

In addition, fungal infection was identified in 6.7% of patients with T1DM-DNI, 11.1% of patients with T2DM-DNI, and 6.8% of patients with non-DM-DNI.

### 3.4. Disease Severity, Therapy, and Prognosis

Table 2 presents the disease severity, treatment method, and prognosis of each DNI group. In terms of disease severity, the white blood cell (WBC) counts in both T1DM-DNI and T2DM-DNI groups were significantly higher than those in the non-DM group (T1DM-DNI vs. T2DM-DNI vs. non-DM-DNI: 14.7 ± 5.8 vs. 13.7 ± 7.5 vs. 13.1 ± 9.4 10^3^/µL, *p* < 0.001). C-reactive protein (CRP) levels were also significantly higher in both T1DM-DNI and T2DM-DNI groups than in the non-DM group (126.4 ± 110.8 vs. 126.4 ± 111.2 vs. 85.1 ± 88.8 mg/dL, *p* < 0.001). In the T1DM-DNI and T2DM-DNI groups, the proportion of patients undergoing tracheostomy was also significantly higher than that in the non-DM-DNI group (5.5% vs. 6.5% vs. 3.7%, *p* < 0.001). Patients in both T1DM-DNI and T2DM-DNI groups had significantly longer durations of hospital stay (16.2 ± 13.3 vs. 15.9 ± 15.4 vs. 11.6 ± 23.2 days, *p* < 0.001) and more ICU admission (12.3% vs. 12.6% vs. 6.8%, *p* < 0.001). In addition, the complication of mediastinitis occurred significantly higher in both the T1DM-DNI and T2DM-DNI than the non-DM-DNI (4.1% vs. 4.7% vs. 2.0%, *p* < 0.001).

Among the three groups of DNI, the proportion of patients with T1DM-DNI and T2DM-DNI undergoing surgical treatment was higher than that of patients with non-DM-DNI (35.6% vs. 30.8% vs. 17.2%, *p* < 0.001). The mortality rates of both the T1DM-DNI and T2DM-DNI groups were significantly higher than the non-DM-DNI group (4.1% vs. 5.5% vs. 3.1%, *p* < 0.001).

### 3.5. Odds Ratios of Mortality for Various Variables in DNI

Table 3 shows the case-control analysis to evaluate the Odds ratios (ORs) of various variables to death. The results show that surgery had a protective effect (adjusted OR of mortality for surgery: 0.21 (95% confidence interval, CI: 0.13–0.34), *p* <0.001); there was no statistically significance of death risk on whether patients underwent tracheostomy or not (adjusted OR of mortality for tracheostomy: 1.62 (0.90–2.90), *p* = 0.105); the need for ICU care had a significant risk of death (adjusted OR of mortality for ICU care: 10.05 (6.91–14.61), *p* <0.001); concurrent mediastinitis significantly increased the risk of death (adjusted OR of mortality for mediastinitis: 2.72 (1.39–5.31), *p* = 0.003).

### 3.6. Survival vs. Death in T1DM-DNI

Table 4 presents the difference in relevant data between the survival and death of patients with T1DM-DNI after receiving treatment. The results showed that there were no significant differences between the two groups in age (survival vs. death: 39.7 ± 30.6 vs. 51.5 ± 17.8 years old, *p* = 0.428), hemoglobin A1c level (10.5 ± 0.2 vs. 9.9 ± 2.6, *p* = 0.614), CRP level (283.9 ± 4.1 vs. 120.8 ± 108.6, *p* = 0.052), bacteremia (33.3% vs. 12.9%, *p* = 0.361), rate of surgical debridement (66.7% vs. 34.3%, *p* = 0.287), tracheostomy (0% vs. 5.71%), length of hospital stay (11.7 ± 7.6 vs. 16.4 ± 13.5 days, *p* = 0.551), and mediastinal infection (33.3% vs. 2.86%, *p* = 0.120). However, the death group had a significantly high WBC count (20.4 ± 0.5 vs. 14.4 ± 5.8 10^3^/µL, *p* = 0.045) and proportion of neutrophil band forms (13.3 ± 9.1 vs. 3.8 ± 4.7%, *p* = 0.020).

### 3.7. Survival vs. Death in T2DM-DNI

Table 5 presents the difference in relevant data between the survival and death of patients with T2DM-DNI after receiving treatment. The results showed that there were no significant differences between the two groups in hemoglobin A1c level (survival vs. death: 8.0 ± 2.9 vs. 8.3 ± 2.4, *p* = 0.377) and tracheostomy (10.1% vs. 6.3%, *p* = 0.116). The death group had significantly high age (63.0 ± 13.2 vs 60.1 ± 13.3 years, *p* = 0.022), WBC count (23.4 ± 16.2 vs. 13.1 ± 6.2 10^3^/µL, *p* < 0.001), proportion of neutrophil band forms (10.1 ± 9.5 vs. 4.0 ± 5.6%, *p* < 0.001), CRP level (199.9 ± 118.1 vs. 121.3 ± 109.0, *p* < 0.001), length of hospital stay (19.0 ± 15.5 vs. 15.7 ± 15.4 days, *p* = 0.029), bacteremia (20.2% vs. 8.1%, *p* < 0.001), and mediastinal infection (11.9% vs. 4.3%, *p* < 0.001). However, the death group had a significantly low rate of surgical debridement (16.5% vs. 31.6%, *p* < 0.001)

## 4. Discussion

DM has been considered a definite predisposing systemic disease in 17–34% of patients with DNI [7,13,14,15,16]. A comprehensive understanding of the bacterial spectrum is essential for precisely choosing effective empirical antibiotics during DNI treatment. The study carried out using the Chang Gung multi-institutional database investigated valuable information for the clinical management of DM-DNI. In our study, the leading pathogens of facultative anaerobe and anaerobe in non-DM-DNI patients were VS and *Prevotella* sp., which is consistent with the results of several previous studies [7,17,18], indicating that the results presented by our control group are consistent with those of previous studies; on this basis, the bacterial spectra of T1DM-DNI and T2DM-DNI were highly reliable.

In our study, the major isolated facultative anaerobic or aerobic bacteria in T2DM-DNI was KP, followed by VS. This finding is in line with the results of previous epidemiologic studies carried out in Asia [7,8]. The main isolated facultative anaerobic or aerobic bacteria in T1DM-DNI were also KP, followed by VS. To the best of our knowledge, this is the first study to investigate the facultative anaerobic or aerobic pathogens involved in T1DM-DNI (Figure 2b). In terms of the isolated “species,” the major species in T2DM-DNI was *Streptococcus* sp., which was a tendency observed for non-DM-DNI; however, the major species in T1DM-DNI was *Klebsiella* sp., which indicated that Klebsiella infection plays a more essential role in T1DM-DNI (Figure 2a).

The most isolated anaerobic bacterial strain in T1DM-DNI is PM and then PI, followed by PA. To the best of our knowledge, this is the first study to investigate the anaerobic pathogens in patients with T1DM with DNI. The order of the main anaerobic bacteria was the same in T2DM-DNI and non-DM-DNI (Figure 3b). Even in terms of species,” the anaerobic bacterial species of these three groups were similar (Figure 3a).

Our previous study demonstrated that patients with T1DM had a 10-fold greater risk for DNI than those without DM [3]. To further discover the pathogen in T1DM-DNI might influence the clinical use of empirical antibiotics. However, the determination of the amount of positive impact it would have on the selection of antibiotics for the management of DNI requires prospective studies for further investigation.

In our study, the positive bacterial culture rate ranged from 42.6% to 68.2% and the culture latency was within 1 week. However, an accurate choice of antibiotics is certainly essential for intensive DNI and might even influence the prognosis of the patients. KP played a significant role in patients with DM with DNI, especially in T1DM (KP: 40.0% for T1DM-DNI; 32.2% for T1DM-DNI; 9.8% for non-DM-DNI). In the past, several studies have explored the association between DM and KP infection in liver abscesses [19,20]. T2DM was found not to impact the neutrophil-killing ability of KP; however, it could reduce cytokine and chemokine production and the intracellular killing ability of other peripheral blood mononuclear cells [19]. Uncontrolled HbA1c levels were also found to render patients with DM susceptible to KP infection [20]. Also, increased oropharyngeal colonization by Gram-negative bacilli was noted in patients with DM by a previous study [21]. However, the pathogenesis of the association between DM and KP is still not clear, and further research is required for investigation.

The results of the present study were consistent with those of previous studies in that the clinical course of DNI with DM as a comorbidity was more severe and had a poorer prognosis [1,2]. In our study, both patients with T1DM and T2DM had more aggressive disease manifestation compared with patients with non-DM, including higher infection marker levels (CBC and CRP levels), more ICU admission, longer hospital stay, more acceptance of surgical debridement and tracheostomy, and more mediastinal complications.

In general, DNI could cause 1–2.5% mortality [22,23,24] and 1.5–6% in patients with medical comorbidities, such as DM [13,16,24]. In our study, the mortality rates were 4.1% in T1DM-DNI, 5.5% in T2DM-DNI, and 3.1% in non-DM-DNI, which are consistent with the previously reported data. In addition, the mortality rate of T1DM-DNI in our study was also close to our previous analysis of the mortality rate (4.8%) of T1DM-DNI using a national database in Taiwan [3].

Furthermore, we divided the T1DM-DNI cases into two groups according to the treatment results, survival and death, and compared the differences (Table 3). Compared with patients in the survival subgroup, those in the death subgroup had significantly higher WBC counts (20.4 ± 0.5 vs. 14.4 ± 5.8 10^3^/ L, *p* = 0.045) and proportions of neutrophil band forms (13.3 ± 9.1 vs. 3.8 ± 4.7%, *p* = 0.02) and non-significantly higher CRP levels (283.9 ± 4.1 vs. 120.8 ± 108.6, *p* = 0.05). Although the rate of surgical debridement was higher in the death subgroup, the difference between the rates of debridement did not attain statistical difference (death vs. survival: 66.67% vs. 37.14%, *p* = 0.55); however, interestingly, 5.71% of patients in the survival subgroup underwent tracheostomy; however, no one in the death subgroup underwent the procedure. In addition, the death subgroup had a non-significantly higher rate of mediastinal complications (mediastinitis of the death group vs. mediastinitis of the survival group: 33.3% vs. 2.86%, *p* = 0.12). The prevalence of bacteremia in the death subgroup was higher than that in the survival group; however, the difference between the two prevalence values was not statistically significant (33.33% vs. 12.86%, *p* = 0.36). On the basis of the aforementioned findings, in patients with T1DM-DNI, clinicians should pay more attention to high serum levels of infection markers (WBC, neutrophils band form, and CRP) and the development of bacteremia and mediastinal complications, which would indicate a potential for mortality. Whether or not tracheostomy could reduce the mortality rate of patients with T1DM-DNI is a topic worthy of further investigation.

Our study had several advantages. First, this database contains a large amount of data from multiple medical centers in Taiwan. Past validation studies have also confirmed that this database comprises >14% inpatient coverage of Taiwan;^9^ therefore, the results of this study could present a real-world situation. Furthermore, this is the first study to investigate the bacterial spectrum in DNI patients with DM. The findings of this study will provide clinicians with an important reference when choosing antibiotics. However, there were some limitations in our study. In general, the pathogenic bacteria responsible for DNI are diverse, and we summarized and analyzed the results of the bacterial cultures to calculate the proportion of each pathogen in the T1DM-DNI, T2DM-DNI, and non-DM-DNI groups; however, the actual state of bacterial culture was more complicated. In addition, although the proportion of those patients with DNI with bacterial cultures ranged from 76.2% to 90.4%, the culture-positive rate was only 42.6%–68.2%; therefore, the bacterial analysis could not represent a complete bacterial spectrum of DNI. Although we used the big-data for research, the control group (non-DM-DNI) had enough samples, but the numbers of patients in T1DM-DNI and T2DM-DNI were not enough to match with the control group on age, gender and selected comorbidities to reduce the difference in the demographic characteristics between the three groups, and this research limitation could cause potential bias. Furthermore, in order to have a comprehensive understanding of the relationship between pathogenic bacteria and the diabetic diseases, collecting pathogenic bacteria and blood samples from patients with T1DM-DNI, T2DM-DNI and non-DM-DNI for multi-omics analysis is a research worth investing in in the future.

## 5. Conclusions

DM-DNI is associated with more severe disease and would cause higher mortality than non-DM-DNI. KP is the leading pathogen not only for T2DM-DNI (32%) but also, and even more, for T1DM-DNI (40%). In addition, the clinicians should pay attention to high WBC counts, CRP levels and proportions of neutrophil band forms, development of bacteremia and mediastinitis which might imply a potential of mortality in patients with DM-DNI.

## Figures and Tables

**Table 1:** Demographic characteristics between DNI in T1DM, T2DM, and non-DM.

Variables	T1DM-DNI		T2DM-DNI		Non-DM-DNI		*p*
	N	%	N	%	N	%	
Total	73		1989		6174		
Gender							0.020 *
Male	39	53.4	1290	64.9	4123	66.8	
Female	34	46.6	699	35.1	2051	33.2	
Age							<0.001 *
<65 year-old	51	69.9	1256	63.2	5233	84.8	
≥65 year-old	22	30.1	733	36.9	941	15.2	
Comorbidities							
ESRD	8	11.0	198	10.0	133	2.2	<0.001 *
LC	7	9.6	133	6.7	156	2.5	<0.001 *
Autoimmune	1	1.4	64	3.2	126	2.0	0.010 ^†^
HTN	33	45.2	1078	54.2	997	16.2	<0.001 *
CVA	12	16.4	322	16.2	294	4.8	<0.001 *
CAD	8	11.0	252	12.7	197	3.2	<0.001 *
COPD	7	9.6	159	8.0	262	4.2	<0.001 *

Abbreviations: DNI, deep neck infection; T1DM, type 1 diabetes mellitus; T2DM, type 2 diabetes mellitus; ESRD, end-stage renal disease; LC, liver cirrhosis; Autoimmune, systemic autoimmune diseases; HTN, hypertension; CVA, cerebral vascular accident; CAD, coronary artery disease; COPD, chronic obstructive pulmonary disease. * Pearson’s chi-squared tests ^†^ Fisher Exact Probability test.

**Table 2:** Analysis of DNI severity, treatment, and prognosis in patients with DNI.

Characteristic	T1DM-DNI (N= 73)		T2DM-DNI (N = 1989)		Non-DM-DNI (N = 6174)		*p*
Severity	Mean ± SD		Mean ± SD		Mean ± SD		
WBC (10^3^/uL)	14.7 ± 5.8		13.7 ± 7.5		13.1 ± 9.4		<0.001 ^#^
CRP (mg/L)	126.4 ± 110.8		126.4 ± 111.2		85.1 ± 88.8		<0.001 ^#^
Hospital stay (days)	16.2 ± 13.3		15.9 ± 15.4		11.6 ± 23.2		<0.001 ^#^
	n	%	n	%	n	%	
Tracheostomy	4	5.5	129	6.5	231	3.7	<0.001 ^†^
ICU admission	9	12.3	251	12.6	422	6.8	<0.001 *
Mediastinitis	3	4.1	93	4.7	125	2.0	<0.001 ^†^
**Therapy**							<0.001 *
Non-surgery ^††^	47	64.4	1377	69.2	5115	82.9	
Surgery	26	35.6	612	30.8	1059	17.2	
**Prognosis**							
Mortality	3	4.1	109	5.5	194	3.1	<0.001 ^†^

Abbreviations: DNI, deep neck infection; T1DM, type 1 diabetes mellitus; T2DM, type 2 diabetes mellitus; WBC, white blood cell; CRP, C-reactive protein; SD, standard deviation; ICU, intensive care unit. * Pearson’s chi-squared test; ^†^ Fisher’s exact test; ^#^ Kruskal–Wallis test ^††^ Antibiotic ± Aspiration.

**Table 3:** Odd ratios of mortality for various variables in DNI patients.

Characteristic	Crude OR (95%CI)	*p*	Adjusted OR * (95%CI)	*p*
Surgery				
No	1 [Reference]		1 [Reference]	
Yes	0.53 (0.37–0.75)	<0.001	0.21 (0.13–0.34)	<0.001
Tracheostomy				
No	1 [Reference]		1 [Reference]	
Yes	2.23 (1.42–3.48)	0.001	1.62 (0.90–2.90)	0.105
ICU				
No	1 [Reference]		1 [Reference]	
Yes	7.90 (5.80–10.76)	<0.001	10.05 (6.91–14.61)	<0.001
Mediastinitis				
No	1 [Reference]		1 [Reference]	
Yes	3.97 (2.29–6.87)	<0.001	2.72 (1.39–5.31)	0.003

* Odds ratio (OR) was adjusted for sex, age, surgery, tracheostomy, ICU, mediastinitis and covariates.

**Table 4:** Analysis of characteristics between the death and survival subgroups of T1DM-DNI.

Characteristic	Death (N = 3)		Survival (N = 70)		*p*
	Mean ± SD		Mean ± SD		
Age (years)	39.7 ± 30.6		51.5 ± 17.8		0.428 *
HbA1c (%)	10.5 ± 0.2		9.9 ± 2.6		0.614 *
WBC (10^3^/uL)	20.4 ± 0.5		14.4 ± 5.8		0.045 *
Band form (%)	13.3 ± 9.1		3.8 ± 4.7		0.020 *
CRP (mg/L)	283.9 ± 4.1		120.8 ± 108.6		0.052 *
Hospital (days)	11.7 ± 7.6		16.4 ± 13.5		0.551 *
	n	%	n	%	
Bacteremia	1	33.3	9	12.9	0.361 ^#^
Surgery	2	66.7	24	34.3	0.287 ^#^
Tracheostomy	0	0	4	5.71	-
Mediastinitis	1	33.3	2	2.86	0.120 ^#^

Abbreviations: HbA1c, hemoglobin A1c; WBC, white blood cell; CRP, C reactive protein. * Wilcoxon rank sum test; ^#^ Fisher’s exact test.

**Table 5:** Analysis of characteristics between the death and survival subgroups of T2DM-DNI.

Characteristic	Death (N = 109)		Survival (N = 1880)		*p*
	Mean ± SD		Mean ± SD		
Age (years)	63.0 ± 13.2		60.1 ± 13.3		0.022 *
HbA1c (%)	8.0 ± 2.9		8.3 ± 2.4		0.377 *
WBC (10^3^/uL)	23.4 ± 16.2		13.1 ± 6.2		<0.001 *
Band form (%)	10.1 ± 9.5		4.0 ± 5.6		<0.001 *
CRP (mg/L)	199.9 ± 118.1		121.3 ± 109.0		<0.001 *
Hospital (days)	19.0 ± 15.5		15.7 ± 15.4		0.029 *
	n	%	n	%	
Bacteremia	22	20.2	153	8.1	<0.001 ^#^
Surgery	18	16.5	594	31.6	0.001 ^#^
Tracheostomy	11	10.1	118	6.3	0.116 ^#^
Mediastinitis	13	11.9	80	4.3	<0.001 ^#^

Abbreviations: HbA1c, hemoglobin A1c; WBC, white blood cell; CRP, C reactive protein. * Student’s t tests; ^#^ Pearson’s chi-squared tests.

## Data Availability

The data presented in this study are available on request from the corresponding author.

## Supplementary Materials

The following are available online at https://www.mdpi.com/article/10.3390/microorganisms9102059/s1, Table S1: Bacterial spectrum in T1DM-, T2DM- and non-DM-DNI.
