# Supplementary material for: The Pathogenic Bacteria of Deep Neck Infection in Patients with Type 1 Diabetes, Type 2 Diabetes, and Without Diabetes from Chang Gung Research Database
Source: Microorganisms. 2021 Sep 29;9(10):2059. doi: 10.3390/microorganisms9102059 (PMC8537061; doi:10.3390/microorganisms9102059)
Supplement: Supplementary file 1 [file microorganisms-09-02059-s001.zip › microorganisms-1379144-supplementary.pdf]

Supplementary table. Bacterial spectrum in T1DM-, T2DM- and non-DM-DNI

		T1DM-DNI		T2DM-DNI		NonDM-DN	
		N	%	N	%	N	%
**Total cases**		73		1989		6174	
Total cultures		66	90.4	1588	79.8	4704	76.2
Positive cultures		45	68.2	907	57.1	2004	42.6
**Classification**	**Pathogens**	**n**	**%**	**n**	**%**	**n**	**%**
facultative anaerobic / GPC	**Staphylococcus sp.**	**10**	**22.2**	**223**	**24.6**	**547**	**27.3**
	Staphylococcus aureus	6	13.3	139	15.3	294	14.7
	Methicillin-resistant (MRSA)	2	4.4	53	5.8	142	7.1
	Methicillin-sensitive (MSSA)	4	8.9	86	9.5	152	7.6
	Staphylococcus epidermidis	1	2.2	8	0.9	50	2.5
	Coagulase (-) staphylococcus	3	6.7	62	6.8	175	8.7
	Staphylococcus saccharolyticus			5	0.6	7	0.3
	Staphylococcus haemolyticus			3	0.3	6	0.3
	Staphylococcus capitis			1	0.1	3	0.1
	Staphylococcus lugdunensis			1	0.1	4	0.2
	Staphylococcus saprophyticus			1	0.1	3	0.1
	Staphylococcus hominis			3	0.3	3	0.1
	Staphylococcus caprae					2	0.1
facultative anaerobic / GNB	**Klebsiella sp.**	**19**	**42.2**	**298**	**32.9**	**208**	**10.4**
	Klebsiella pneumoniae	18	40.0	292	32.2	197	9.8
	Klebsiella pneumoniae-ESBL*			2	0.2	2	0.1
	Klebsiella oxytoca	1	2.2	4	0.4	9	0.4
	Klebsiella oxytoca-ESBL						
facultative anaerobic / GPC	**Streptococcus sp.**	**18**	**40.0**	**337**	**37.2**	**1088**	**54.3**
	Viridans Streptococcus	10	22.2	211	23.3	694	34.6
	Streptococcus agalactiae	1	2.2	18	2.0	44	2.2
	Beta-Streptococcus nonABD	2	4.4	40	4.4	170	8.5
	Beta-Streptococcus group D	3	6.7	11	1.2	26	1.3
	Streptococcus pneumoniae					6	0.3
	Streptococcus anginosus	1	2.2	15	1.7	29	1.4
	Streptococcus constellatus	1	2.2	25	2.8	64	3.2
	Streptococcus oralis			3	0.3	9	0.4

	Streptococcus gordonii			1	0.1	2	0.1
	Streptococcus mitis			8	0.9	17	0.8
	Streptococcus intermedius			5	0.6	13	0.6
	Streptococcus salivarius					9	0.4
	Streptococcus sanguinis					3	0.1
	Streptococcus vestibularis					1	0.0
	Streptococcus sp.					1	0.0
facultative anaerobic / GPC	**Enterococcus sp.**	**1**	**2.2**	**23**	**2.5**	**52**	**2.6**
	Enterococcus faecalis	1	2.2	14	1.5	41	2.0
	Enterococcus faecium			2	0.2	3	0.1
	Enterococcus avium			1	0.1	2	0.1
	Enterococcus durans			1	0.1		
	Enterococcus raffinosus			3	0.3		
	Enterococcus sp.			2	0.2	6	0.3
facultative anaerobic / GPC	**Morbillorum sp.**					**3**	**0.1**
facultative anaerobic / GNB	**Enterobacter sp.**	**1**	**2.2**	**21**	**2.3**	**50**	**2.5**
	Enterobacter aerogenes			5	0.6	7	0.3
	Enterobacter cloacae	1	2.2	13	1.4	39	1.9
	Enterobacter agglomerans			1	0.1		
	Enterobacter gergoviae			1	0.1	2	0.1
	Enterobacter sp.			1	0.1	2	0.1
facultative anaerobic / GNB	**Escherichia coli**	**1**	**2.2**	**25**	**2.8**	**42**	**2.1**
	Escherichia coli	1	2.2	21	2.3	40	2.0
	Escherichia coli-ESBL			4	0.4	2	0.1
facultative anaerobic / GNB	**Proteus sp.**			**11**	**1.2**	**39**	**1.9**
	Proteus mirabilis			11	1.2	29	1.4
	Proteus penneri					3	0.1
	Proteus vulgaris					7	0.3
facultative anaerobic / GNB	**Haemophilus sp.**			**1**	**0.1**	**8**	**0.4**
	Haemophilus influenzae					3	0.1
	Haemophilus parainfluenzae			1	0.1	3	0.1
	haemophilus sp.					2	0.1

facultative anaerobic / GNB	**Salmonella sp.**	**1**	**2.2**	**12**	**1.3**	**2**	**0.1**
	Salmonella enterica group B			4	0.4		
	Salmonella enterica group D	1	2.2	7	0.8	2	0.1
	Salmonella choleraesuis			1	0.1		
facultative anaerobic / GNB	**Eikenella corrodens**	**3**	**6.7**	**18**	**2.0**	**77**	**3.8**
facultative anaerobic / GNB	**Citrobacter sp.**	**1**	**2.2**	**10**	**1.1**	**34**	**1.7**
	Citrobacter freundii	1	2.2	2	0.2	10	0.5
	Citrobacter amalonaticus			1	0.1	1	0.0
	Citrobacter diversus			6	0.7	23	1.1
	Clostridium sp.			1	0.1		
facultative anaerobic / GPB	**Corynebacterium sp.**			**13**	**1.4**	**27**	**1.3**
	Corynebacterium jeikeium			4	0.4	2	0.1
	Corynebacterium urealyticum					1	0.0
	Corynebacterium sp.			9	1.0	24	1.2
facultative anaerobic / GNB	**Serratia marcescens**			**6**	**0.7**	**26**	**1.3**
facultative anaerobic / GPB	**Actinomyces sp.**			**5**	**0.6**	**17**	**0.8**
	actinomyces israelii					1	0.0
	actinomyces sp.			5	0.6	16	0.8
facultative anaerobic / GPB	**Lactobacillus**			**3**	**0.3**	**12**	**0.6**
facultative anaerobic / GNB	**Providencia sp.**			**2**	**0.2**	**1**	**0.0**
	Providencia stuartii			2	0.2		
	Providencia rettgeri					1	0.0
facultative anaerobic / GNB	**Aeromonas sp.**			**1**	**0.1**	**3**	**0.1**
aerobic / GNB	**Pseudomonas sp.**			**60**	**6.6**	**141**	**7.0**
	Pseudomonas aeruginosa			55	6.1	126	6.3
	Pseudomonas aeruginosa-CR[#]					1	0.0
	Pseudomonas putida			1	0.1	1	0.0
	Pseudomonas stutzeri					1	0.0

	Pseudomonas cepacia			1	0.1	2	0.1
	Pseudomonas pseudomallei			1	0.1		
	Pseudomonas diminuta					1	0.0
	Pseudomonas vesicularis					1	0.0
	Pseudomonas oryzihabitans					1	0.0
	Pseudomonas putrefaciens					2	0.1
	Pseudomonas paucimobilis					1	0.0
	Pseudomonas sp.			2	0.2	4	0.2
aerobic / GNB	**Acinetobacter sp.**	**2**	**4.4**	**16**	**1.8**	**35**	**1.7**
	Acinetobacter baumannii	2	4.4	9	1.0	21	1.0
	Acinetobacter baumannii-PDR†			1	0.1		
	Acinetobacter baumannii-MDR‡			1	0.1	4	0.2
	Acinetobacter lwoffii			2	0.2	3	0.1
	Acinetobacter nosocomialis			1	0.1		
	Acinetobacter junii			1	0.1	1	0.0
	Acinetobacter haemolyticus					1	0.0
	Acinetobacter pittii					1	0.0
	Acinetobacter sp.			1	0.1	4	0.2
aerobic / GNC	**Neisseria sp.**	**1**	**2.2**	**21**	**2.3**	**129**	**6.4**
	Neisseria sicca			2	0.2	1	0.0
	Neisseria subflava			1	0.1	4	0.2
	Neisseria mucosa					1	0.0
	Neisseria sp.	1	2.2	18	2.0	123	6.1
aerobic / GPC	**Stomatococcus**			**6**	**0.7**	**26**	**1.3**
aerobic / GNB	**Stenotrophomonas maltophilia**					**5**	**0.2**
aerobic / GPC	**Micrococcus**			**4**	**0.4**	**14**	**0.7**
aerobic / GNB	**Chryseobacterium sp.**					**5**	0.2
	Chryseobacterium indologenes					4	0.2
	Chryseobacterium meningosepticum					1	0.0
aerobic / GNB	**Moraxella sp.**			**11**	**1.2**	**22**	**1.1**
	Moraxella catarrhalis			1	0.1		
	Moraxella morganii			10	1.1	19	0.9
	Moraxella osloensis					3	0.1
aerobic /GPB	**Mycobacterium tuberculosis complex**					**3**	0.1
aerobic /GPB	**Nontuberculous Mycobacterium**					**2**	0.1
anaerobic / GPC	**Peptostreptococcus sp.**	**11**	**24.4**	**185**	**20.4**	**686**	**34.2**
	Peptostreptococcus micros	6	13.3	57	6.3	240	12.0

	Peptostreptococcus anaerobius	3	6.7	29	3.2	78	3.9
	Peptostreptococcus magnus			4	0.4	30	1.5
	Peptostreptococcus asaccharolyticus	1	2.2	5	0.6	12	0.6
	Peptostreptococcus prevotii			1	0.1		
	Peptostreptococcus intermedius					1	0.0
	Peptostreptococcus sp.	1	2.2	89	9.8	325	16.2
anaerobic / GNB	**Prevotella sp.**	**13**	**28.9**	**203**	**22.4**	**660**	**32.9**
	Prevotella disiens			1	0.1	4	0.2
	Prevotella bivia			4	0.4	9	0.4
	Prevotella buccae	1	2.2	19	2.1	40	2.0
	Prevotella intermedia	5	11.1	35	3.9	178	8.9
	Prevotella melaninogenica	2	4.4	23	2.5	72	3.6
	Prevotella denticola			2	0.2	9	0.4
	Prevotella oralis			1	0.1	3	0.1
	Prevotella oris			3	0.3	5	0.2
	Prevotella heparinolytica			1	0.1	2	0.1
	Prevotella corporis					1	0.0
	Prevotella loescheii					1	0.0
	Prevotella sp.	5	11.1	114	12.6	336	16.8
anaerobic / GNC	**Veillonella sp.**	**3**	**6.7**	**47**	**5.2**	**174**	**8.7**
	Veillonella dispar			10	1.1	22	1.1
	Veillonella parvula			8	0.9	19	0.9
	Veillonella alcalescens					1	0.0
	Veillonella sp.	3	6.7	29	3.2	132	6.6
anaerobic / GNB	**Fusobacter sp.**	**3**	**6.7**	**40**	**4.4**	**124**	**6.2**
	Fusobacter nucleatum	3	6.7	25	2.8	71	3.5
	Fusobacter necrophorum			7	0.8	20	1.0
	Fusobacter varium			1	0.1	4	0.2
	Fusobacter sp.			7	0.8	29	1.4
anaerobic / GNB	**Bacteroides sp.**			**22**	**2.4**	**62**	**3.1**
	Bacteroides thetaiotaomicron			3	0.3	12	0.6
	Bacteroides fragilis			11	1.2	19	0.9
	Bacteroides gracilis			1	0.1	2	0.1
	Bacteroides ovatus			1	0.1	1	0.0
	Bacteroides uniformis					1	0.0
	Bacteroides ureolyticus					2	0.1
	Bacteroides vulgatus					2	0.1

	Bacteroides capillosus					1	0.0
	Bacteroides sp.			6	0.7	22	1.1
anaerobic / GPB	**Propionibacterium sp.**			**18**	**2.0**	**82**	**4.1**
	Propionibacterium acnes			14	1.5	56	2.8
	Propionibacterium avidum				0.0	3	0.1
	Propionibacterium propionicus			1	0.1		
	Propionibacterium granulosum					1	0.0
	Propionibacterium sp.			3	0.3	22	1.1
anaerobic / GPB	**Clostridium sp.**					**13**	**0.6**
	Clostridium bifermentans					1	0.0
	Clostridium perfringens					2	0.1
	Clostridium sordellii					1	0.0
	Clostridium sp.					9	0.4
anaerobic / GNC	**Porphyromonas sp.**					11	0.5
anaerobic / GNB	**Capnocytophaga sp.**			**1**	**0.1**	**8**	**0.4**
	Capnocytophaga sputigena			1	0.1		
	Capnocytophaga sp.			1	0.1	8	
fungus	**Yeast-like**	**3**	**6.7**	**68**	**7.5**	**85**	**4.2**
	**Mold**			**1**	**0.1**	**1**	**0.0**
	**Candida sp**			**13**	**1.4**	**15**	**0.7**
	Candida albicans			13	1.4	10	0.5
	Candida glabrata					3	0.1
	Candida tropicalis					2	0.1
	**Penicillium marneffei**					**1**	**0.0**
	**Unknown GPB**			**4**	**0.4**	**15**	**0.7**
	**Unknown aerobes**			**2**	**0.2**	**5**	**0.2**

[*]ESBL, extended-spectrum β-lactamases; [#]CR, Carbapenem-resistant; [†]PDR, pandrug-resistant; [‡]MDR, multidrug-resistant
